# Life cycle impact assessment and life cycle cost assessment for centralized and decentralized wastewater treatment plants in Thailand

**DOI:** 10.1038/s41598-022-18852-y

**Published:** 2022-08-25

**Authors:** Rutjaya Prateep Na Talang, Sanya Sirivithayapakorn, Sucheela Polruang

**Affiliations:** Department of Environmental Engineering, Faculty of Engineering, Kasetsart University, Bangkok, 10900 Thailand

**Keywords:** Environmental impact, Climate-change impacts, Environmental economics, Sustainability

## Abstract

This research investigates the cost-effectiveness of four sludge treatment scenarios for centralized (C) and decentralized (D) wastewater treatment plants (WWTP) using life cycle cost assessment (LCCA). The environmental impacts and costs are quantified by Stepwise2006. The most environmentally and financially viable WWTP construction option for Bangkok, Thailand (2022–2031) is determined in terms of LCCA and net present value (NPV). The environmental costs of D-treatment scenarios are lower than those of C-treatment scenarios. The total environmental costs of C- and D-fertilizer scenarios are lower than those of C- and D-dewatering scenarios. The net cash flow per functional unit of C-WWTPs is higher than that of D-WWTPs. The C-fertilizer scenario is the most environmentally and economically viable treatment scenario due to the lowest LCCA deficit (−5.58 THB_2020_ per m^3^ treated effluent). Composting should thus be adopted for treating sludge. The most environmentally and financially viable WWTP construction option is option I (building four C-WWTPs within 10 years) due to the lowest LCCA deficit (−19925 million THB_2020_) and smallest financial loss (NPV = −6309.96 million THB_2020_). Essentially, the local administration of the capital should adopt option I as a guideline in formulating the wastewater treatment management policy of 2022–2031.

## Introduction

Rapid population growth and urbanization contribute to increasing demand for wastewater collection and treatment. In urbanized areas, household wastewater is collected and treated at a centralized (C) or decentralized (D) wastewater treatment plant (WWTP). The C-wastewater management typically involves extensive sewer networks, complex and efficient wastewater collection system, standard treatment technology, and high treatment efficiency. Meanwhile, in the D-wastewater management, household wastewater is collected and treated close to the source using modular subsystems, rendering the construction of complex sewer networks unnecessary which in turn enhances the system flexibility^1^.

A number of factors influence the investment decision between C- and D-wastewater management systems, e.g., sewerage network supply, land-use opportunity, availability of skilled staff, and financial and technical capability^2^. As a result, in many developing countries, given the financial constraint, the D-wastewater management is regarded as an economically viable alternative to the C-wastewater management.

The construction and operation costs of D-wastewater treatment systems vary greatly, depending on the number and the layout of modular subsystems. Besides, the total cost of the D-treatment system equipped with large modular subsystems is generally lower than that of the C-wastewater treatment system, due to lower operation and maintenance needs of the D-treatment system. In addition, the well-designed D-modular subsystems possess a cost advantage over the C-wastewater management^3^.

Life cycle thinking focuses on the environmental and socio-economic impacts of a product or service through the entire lifecycle^4^. Life cycle assessment (LCA) normally focuses on environmental impacts e.g., human toxicity, ecotoxicity, global warming, eutrophication and resource depletion, consisting of four steps: (1) definition of system boundary, functional unit and assumptions, (2) life cycle inventory (LCI), (3) life cycle impact assessment (LCIA), and (4) interpretation^5,6^. For the economic impact, life cycle cost (LCC) takes into account the net cash flow i.e., sources of revenues and expenditures while, life cycle cost assessment (LCCA) takes into account LCC and environmental costs^7^.

Existing LCA studies that incorporate the life cycle cost (LCC) concept are listed in Table 1. Essentially, the existing studies focus primarily on the centralized wastewater treatment systems, e.g., Awad et al.^8^, Tabesh et al.^9^, Polruang et al.^10^, Bertanza et al.^11^. Meanwhile, Lorenzo-Toja et al.^12^, Lorenzo-Toja et al.^13^ investigated both C- and D-wastewater treatment systems in terms of LCA and LCC.

**Table 1:** Existing literature on LCA and LCC of centralized and decentralized WWTPs.

Source	Location	Data collection period	Type of WWTP		Wastewater treatment process	Assessment			Results
			CWWTP	DWWTP		LCA	LCC	LCCA	
This study	Bangkok, Thailand	2016–2017	✔	✔	AS	✔	✔	✔	The C-fertilizer scenario has the highest LCCA and is the most environmentally and economically viable treatment scenario because of the highest net cash flow
Hospido et al. ^14^	Galicia, Spain	2000–2001	✔		AS	✔	–	–	The main contributors to the environmental impacts were water discharge and sludge application to land. The AS achieved high nitrogen removal efficiency
Rodriguez-Garcia et al.^15^	Spain	2008	✔		AS, Extended aeration and Oxidation ditch	✔	✔	–	WWTPs with organic removal technology were less costly in environmental and economic terms. However, improvement in effluent worsened global warming impact and increased overall expense
Ontiveros and Campanella ^16^	Argentina	N.A. (simulation data)		✔	Conventional and biological nutrient removal processes	✔	–	–	The nutrient removal system significantly improved the quality of effluent and biosolids while reducing overall energy consumption
Li et al.^17^	Kunshan, China	2010	✔		Anaerobic-Anoxic–Oxic	✔	–	–	Eutrophication, global warming, and waterborne suspended particles were major contributing factors to the environmental impacts of Kunshan WWTP
Yoshida et al.^18^	Copenhagen, Denmark	2011	✔		Biological nitrogen removal	✔	–	–	Data collection should be standardized and expanded to include energy and chemical usage data, the elimination of a reporting threshold, the expansion of substance coverage, and the inclusion of non-point fugitive gas emissions
Lorenzo-Toja et al.^13^	Spain	2011	✔	✔	N.A	✔	–	–	The research examined 113 WWTPs with organic matter and nutrient removal technology in Spain using LCA. The results showed that large WWTPs were highly efficient
Lorenzo-Toja et al.^12^	Spain	2011	✔	✔	N.A	✔	✔	–	The research studied 22 WWTPs in Spain using LCA and LCC; and reported that the operation and maintenance costs of WWTPs with phosphorus and/or nitrogen removal were greater than those without nutrient removal
Limphitakphong et al.^19^	Bangkok, Thailand	2008	✔		Contact stabilization, AS with nutrient removal, Cyclic AS, Two-stage AS, and Vertical loop reactor AS	✔	✔	–	Vertical loop reactor AS achieved the highest pollutant removal efficiency. Electricity consumption was the main contributor of global warming potential and operation cost, while eutrophication as nutrient discharge was the major contributor of total impacts
Bertanza et al.^11^	Brescia-Verziano, Italy	N.A	✔		Conventional AS and Membrane bioreactor	✔	✔	–	The advantages of conventional AS are ease of operation, minimal cost, and low energy consumption, while membrane bioreactor technology is commonly used with similar overall environmental footprint to AS
Garfí et al.^20^	Catalonia and Barcelona, Spain	N.A		✔	AS, Hybrid constructed wetland and High rate algal pond	✔	✔	–	The AS process was costliest (in financial terms) among three treatment schemes under study. Meanwhile, wetland system required vast plots of land
Polruang et al.^10^	Bangkok, Thailand	2014	✔		Contact stabilization, AS with nutrient removal, AS, Two-stage AS, and Vertical loop reactor AS	✔	–	–	Electricity consumption was the main contributor to almost all environmental impacts. The reduction in fossil fuels use for electricity production reduced global warming, abiotic depletion, and acidification impacts
Arashiro et al.^21^	Barcelona, Spain	N.A		✔	AS and High rate algal pond	✔	✔	–	High rate algal pond system with biogas and biofertilizer production was more environmentally friendly than AS system. The high rate algal pond system was the sustainable and cost-effective technology for wastewater treatment in small communities
Singh et al.^22^	India	2014–2015		✔	Moving bed biofilm reactor	✔	✔	–	Moving bed biofilm reactor achieved high organic removal efficiency but low nutrient removal efficiency
Awad et al.^8^	Gamasa, Egypt	2010	✔		Conventional AS	✔	✔	–	The operation phase generated greater environmental impacts than the construction phase. Air emissions and energy consumption were the main contributors of environmental impacts
Tabesh et al.^9^	Tehran, Iran		✔		Aeration lagoons	✔	–	–	Use of biogas contributed significantly to the environmental impacts. Use of treated wastewater as irrigating water on farmland mitigated the eutrophication effect

**Remark: **N.A. denotes no available data.

**Note:** The capacity of centralized WWTPs is more than 2500 m^3^ per day.

However, there exists no research that comparatively investigates the C- and D-wastewater treatment systems by using LCCA. This current research is thus the first that applies the LCCA concept to comparatively investigate the C- and D-wastewater management.

Specifically, the aims of this research are: (1) to comparatively investigate the environmental impacts and costs by LCA and the cost-effectiveness by LCCA of the C- and D-biological wastewater treatment systems under four sludge treatment scenarios (C-dewatering, C-fertilizer, D-dewatering, D-fertilizer); and (2) to determine the most cost-effective sludge treatment scenario with the lowest environmental impacts and highest net cash flow. Furthermore, this study also determines the most environmentally and financially optimal WWTP construction option for Thailand’s capital Bangkok between 2022 and 2031 with respect to the LCCA and net present value.

## Materials and methods

**Figure 1:**
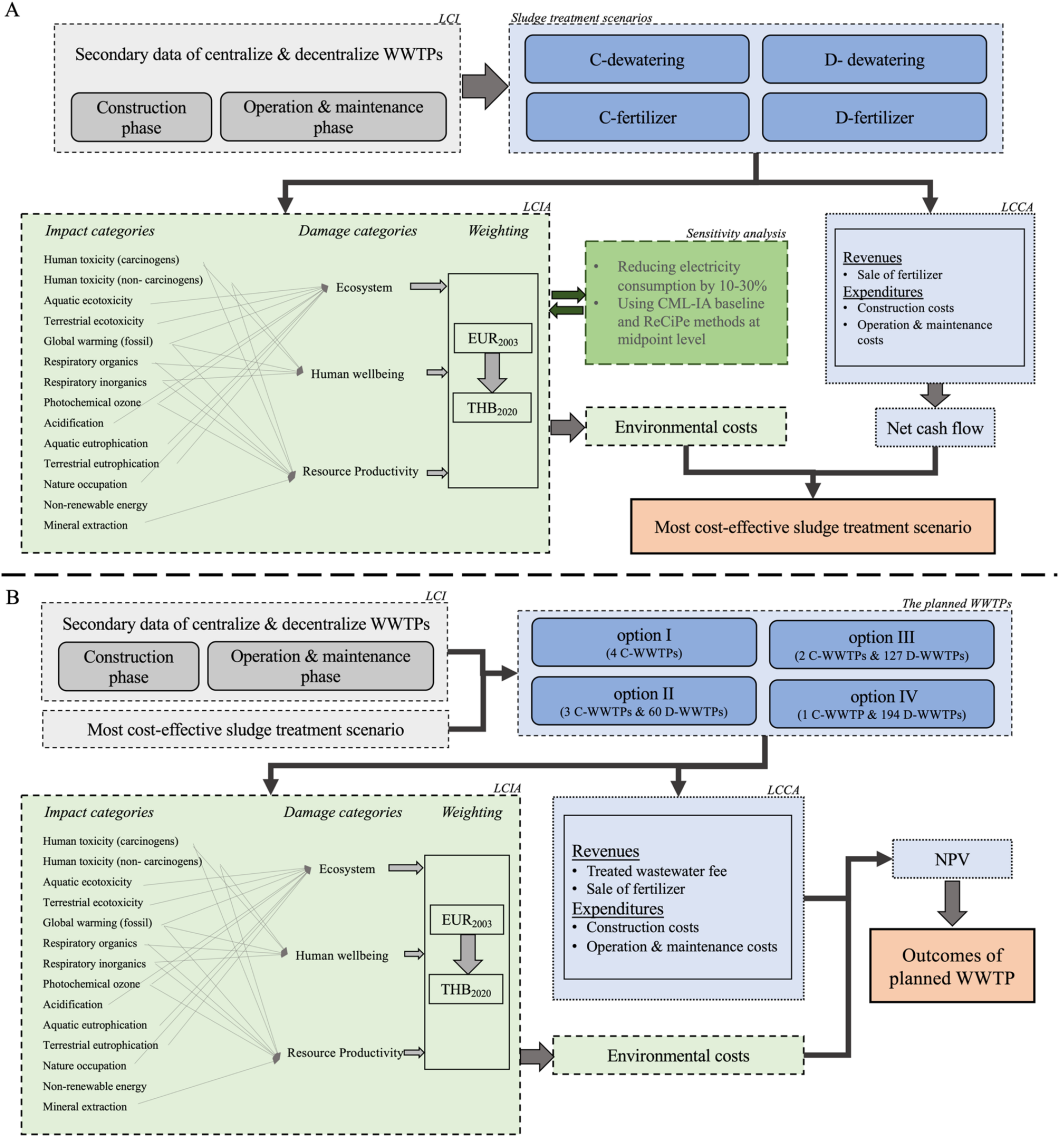
The overall research framework and methodology of (A.) four sludge treatment scenarios and (B.) four WWTP construction options.

Figure 1 shows the overall research framework and methodology of the four sludge treatment scenarios and the four WWTP construction options.

### System boundary and assumptions

The system boundary is of cradle-to-grave life cycle, encompassing the construction, collection and transportation of wastewater by pipeline to WWTPs, treatment operation, system maintenance, and sludge management. The system boundary excludes the plant demolition due to the unavailability of data specific to Thailand. The functional unit (FU) is one cubic meter (m^3^) of treated effluent. The effluent meets the requirements on effluent standards of the country’s regulatory body^23^. Data on the characteristics of influent and effluent belong to the years 2016–2017.

Thailand’s capital Bangkok currently has eight centralized WWTPs (i.e., *Bangsue, Chatuchak, Chongnonsi, Dindaeng, Nongkaem, Rattanakosin, Sipraya,* and *Thungkru* plants), and 12 decentralized WWTPs (i.e., *Bangbua, Bangna, Bonkai, Huai-khwang, Hua-mark, KhlongChan, KhlongToei, RamIntra, RomKlao, ThaSai,* and *Tungsonghong I* and *II*).

**Figure 2:**
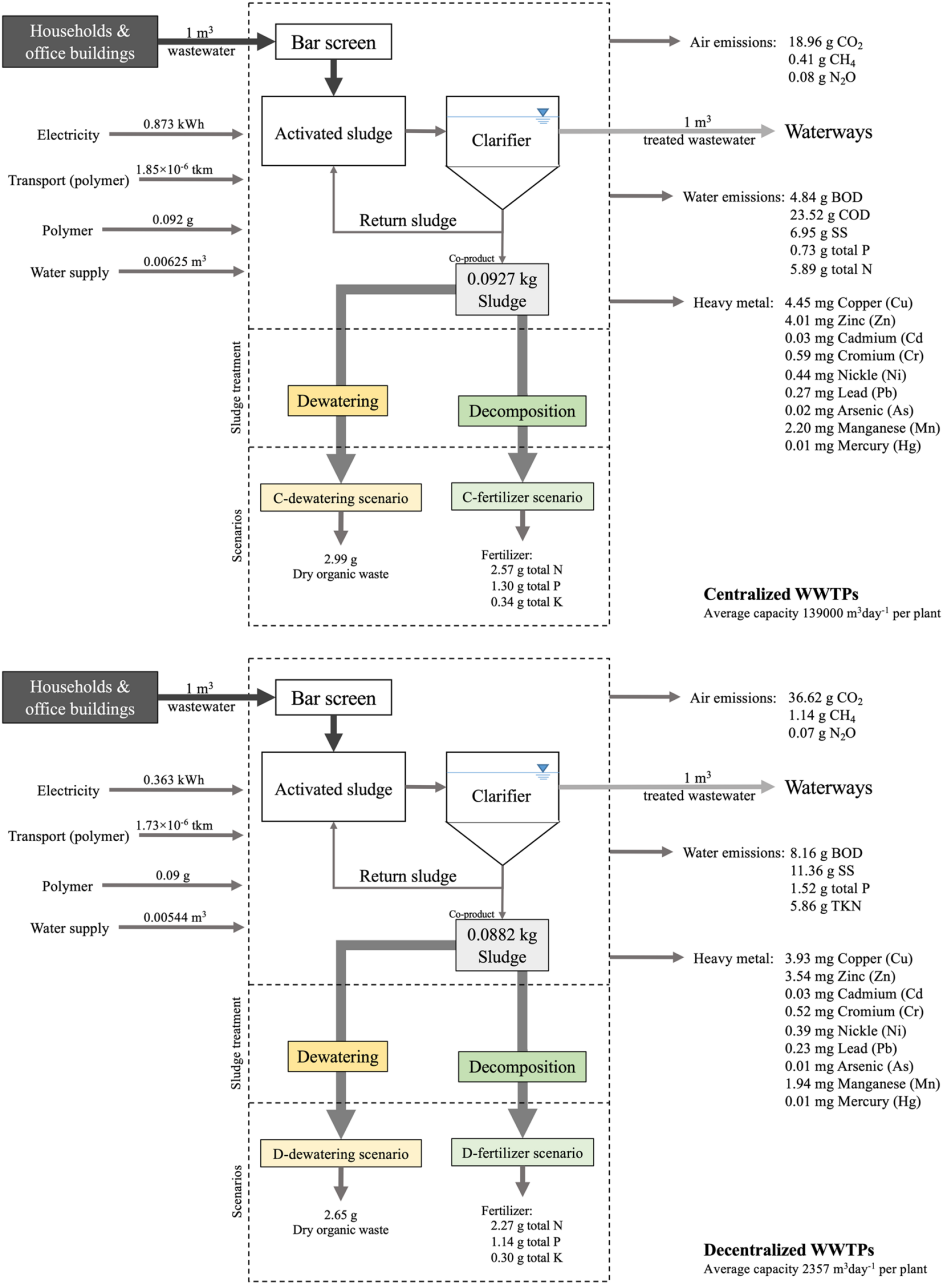
Average inventory data of centralized and decentralized sludge treatment scenarios: C-dewatering, C-fertilizer, D-dewatering and D-fertilizer.

Figure 2 shows two centralized (C) sludge treatment scenarios: C-dewatering and C-fertilizer (decomposition) treatment scenarios and two decentralized (D) sludge treatment scenarios: D-dewatering and D-fertilizer (decomposition) treatment scenarios.

### Life cycle inventory (LCI) and sludge treatment

The 2016–2017 average inventory data of the centralized (i.e., C-dewatering and C-fertilizer) and decentralized sludge treatment scenarios (D-dewatering and D-fertilizer) are also respectively provided in Fig. 2 and Table SI-1 of Supplementary Information (SI)^24^. In the analysis, this study focuses on the existing eight centralized WWTPs and seven (out of 12) decentralized WWTPs due to either the temporary closure of the remaining decentralized WWTPs for renovation or a lack of data. The average capacity of the centralized and decentralized WWTPs are 139,000 and 2357 m^3^ per day, respectively. The useful life of the WWTP and sewer network systems are assumed to 30 years.

The air emissions are calculated following the guidelines of the Intergovernmental Panel on Climate Change and the United States Environmental Protection Agency^25,26^. For the centralized WWTPs, the sludge as co-product from wastewater treatment is anaerobically digested for biogas and decomposed for fertilizer. Meanwhile, for the decentralized WWTPs, the sludge is treated by dewatering for dry organic waste. Due to data unavailability, the operation of anaerobic digestion is excluded from this study.

Dewatering is a mechanical process to separate solid from liquid parts in order to reduce the sludge moisture content^27^. In this study, all the centralized and decentralized WWTPs are equipped with the thickening system to remove the sludge moisture content by up to 3%^27^. After thickening, for the centralized wastewater treatment, sludge is transported by truck to *Nongkeam* WWTP to convert into biogas and fertilizer (i.e., decomposition). Meanwhile, for the decentralized wastewater treatment, sludge is sun-dried and used to fill the land (i.e., dewatering).

In the decomposition, 70% sludge and 30% organic matter are composted by the windrow method to improve the quality of compost^24^. According to Seleiman et al^28^, sludge contains 25.77, 12.98, and 3.40 g of nitrogen, phosphorus, and potassium per kg dry matter.

This current research relies on the LCI consequential modeling. For the dewatering, the sludge is used to fill the land, while the sludge is used as a substitute for chemical fertilizers for the decomposition.

### Life cycle impact assessment (LCIA)

LCI of four sludge treatment scenarios (C-dewatering, C-fertilizer, D-dewatering, D-fertilizer) are assessed the environmental impacts using Stepwise2006 of SimaPro based on the ecoinvent database. Table SI-2 of SI is provided the details of midpoint impact categories in the Stepwise2006 method. This research focuses on fourteen environmental impacts, including human toxicity (carcinogens), human toxicity (non- carcinogens), aquatic ecotoxicity, terrestrial ecotoxicity, global warming (fossil), respiratory organics, respiratory inorganics, photochemical ozone, acidification, aquatic eutrophication, terrestrial eutrophication, nature occupation, non-renewable energy, and mineral extraction. All environmental impacts are grouped into three damage categories i.e., impacts on ecosystem, human well-being and resource depletion. Additionally, the impact on ecosystem is classified as atmospheric, lithospheric and hydrospheric impacts.

The environmental costs are determined by Stepwise monetary weighting factors that detail in Table SI-3 of SI^29,30^ and converted into the year 2020 Thai currency (THB_2020_)^31^ using purchasing power parity (PPP) (i.e., PPP_US$2002_ and PPP_THB2002_) and Thailand’s gross domestic product (GDP) deflator index of 2002 and 2020. The details of currency conversion are provided in Table SI-4 of SI.

### Sensitivity analysis

According to ISO14044:2006^5^, the sensitivity analysis has the goal to assess the reliability of the final outcomes. Firstly, the electricity consumption is the main contributor of environmental impacts^32^, all of the sludge treatment scenarios (C-dewatering, C-fertilizer, D-dewatering, D-fertilizer) are thus assumed to successfully reduce the electricity consumption by 10%, 20%, and 30%. Besides, evidence shows that the choice of LCIA method influences the environmental impact outcomes^33^. As a result, this research also performs the sensitivity analysis of the four sludge treatment scenarios (C-dewatering, C-fertilizer, D-dewatering, D-fertilizer), given 10%, 20%, and 30% reduced electricity consumption, using CML-IA baseline and ReCiPe methods at midpoint level, in addition to Stepwise2006.

### Life cycle cost assessment (LCCA)

In the LCCA, the source of revenue (or cash inflow) is the sale of decomposed sludge fertilizer which is priced at 2 THB/kg. For the expenditures (or cash outflow), the construction costs, including the costs of collection system, treatment plant, and dewatering system, are gleaned from publicly available data and prior publications^34–36^. The operation and maintenance (O&M) costs include the costs of electricity, water supply, chemical reagents, sludge treatment, and administrative overheads, e.g., wage, management fee (Department of Drainage and Sewerage^24^.

The construction and O&M costs are converted into the 2020 Thai baht (THB_2020_) based on the purchasing power parity (PPP) and gross domestic product (GDP) deflator index^31^. The PPP and GDP deflator index are used to reconcile differences between the three currencies (US$, EUR and Thai Baht) and multiple time periods.

The LCCA of four sludge treatment scenarios (C-dewatering, C-fertilizer, D-dewatering, D-fertilizer) entail their respective cash inflow and outflow and the environmental costs. In this research, the most cost-effective sludge treatment scenario possesses the largest LCCA surplus or smallest LCCA deficit.

### The planned WWTPs

The current total capacity of the centralized and decentralized WWTPs in the capital Bangkok is 1,112,000 and 25,000 m^3^ per day, respectively. The new centralized WWTP in *Minburi* district is currently under construction and expected to be complete in 2022, with the maximum wastewater treatment capacity of 10,000 m^3^ per day. In 2021, all the existing WWTPs combined are capable of treating only 68.33% of Bangkok’s municipal wastewater, given the per-capita daily wastewater generation of 0.2 m^3^^37^ and the population of 8.39 million^38^.

By 2027, the population of Thailand’s capital Bangkok is projected to be 8.48 million, with the wastewater generation of around 1.70 million m^3^ per day. According to Department of Drainage and Sewerage^24^,Japan International Cooperation Agency^34^, it takes two years to construct a centralized WWTP at the cost of 3358.27 million THB_2020_; and one year for a decentralized WWTP at the cost of 118.95 million THB_2020_. An annual budget of around 4500 million THB_2020_ is set aside for the construction of new WWTPs (Department of Drainage and Sewerage^24^.

Given the budgetary constraint and capacity limitations of WWTPs, the Bangkok Metropolitan Administration (BMA) should opt for an environmentally and economically optimal number of future centralized and decentralized WWTPs that match the municipal wastewater treatment demand and supply by 2031. Besides, in this research, the environmental and financial costs of the four sludge treatment scenarios (C-dewatering, C-fertilizer, D-dewatering, D-fertilizer) are used to determine the optimal combined number of centralized and decentralized WWTPs to construct (i.e., options I, II, III, and IV).

In finance, net present value (NPV) is used in capital budgeting and investment planning to determine the profitability of an investment project. Mathematically, NPV is the present value of the future cash flows, discounted at the required rate of return, minus the initial investment. In this research, the discount rate or required rate of return is 10%, given that the discount rate of public infrastructure projects in developing countries is around 10%^39^. For the planned WWTPs to be constructed in the capital Bangkok, the sources of revenue are fee from wastewater treatment and sale of decomposed sludge fertilizer, while the expenditures include the O&M and environmental costs, excluding the construction cost since the WWTPs are public infrastructure projects funded from state coffers. The wastewater treatment fee is 2 THB_2020_ per m^3^ wastewater^40^. This study also assumes that the BMA could collect 80% of the treated wastewater fee.

Figure 1 shows the four WWTP construction options for the period of 2022–2031: building four centralized WWTPs (option I), building three centralized and 60 decentralized WWTPs (option II), building two centralized and 127 decentralized WWTPs (option III), and building one centralized and 194 decentralized WWTPs (option IV).

## Results and discussion

### Environmental outcomes and sensitivity analysis

As shown in Fig. 2, the average input of the centralized treatment (C-dewatering and C-fertilizer), including electricity, polymer, transportation of polymer, and water supply, are higher than that of the decentralized treatment (D-dewatering and D-fertilizer). Meanwhile, the average output of the centralized treatment, including air (CO_2_, CH_4_) and water (BOD, total P) emissions, are lower due to higher treatment efficiency of the centralized WWTPs. The direct greenhouse gas (GHG) emissions of the centralized treatment are lower than the decentralized treatment. However, the indirect GHG emissions (i.e., electricity consumption) of the centralized treatment are higher. Higher heavy metals of the centralized treatment scenarios are attributable to higher sludge generation of the centralized treatment than the decentralized treatment.

Table 2 shows the contribution analysis results in terms of the environmental impacts of the four sludge treatment scenarios (C-dewatering, C-fertilizer, D-dewatering, D-fertilizer). Under all treatment scenarios, electricity consumption contributes negatively to almost all environmental impact categories, except for human toxicity (non-carcinogens), aquatic ecotoxicity, and aquatic eutrophication. Human toxicity (non-carcinogens) and aquatic ecotoxicity are inversely correlated to heavy metals in sludge, while aquatic eutrophication is inversely correlated to effluent quality. Electricity consumption of C-dewatering and C-fertilizer is the main contributor of mineral extraction, while the main contributor of mineral extraction of D-dewatering and D-fertilizer is tap water consumption. The mechanical aeration is responsible for the lion’s share of the electricity cost in wastewater treatment^10,16,32^. The electricity consumption of the centralized treatment scenarios (0.873 kWh/m^3^ treated wastewater) is greater than the decentralized treatment scenarios (0.363 kWh/m^3^ treated wastewater). The average electricity consumption of 22 WWTPs in Spain (0.36 kWh/m^3^ treated wastewater)^12^ is lower that both centralized and decentralized treatment scenarios of this study. In comparison with Arashiro et al.^21^, the electricity consumption and sludge of the decentralized treatment in this study is lower. All of the environmental impacts, excluding aquatic eutrophication, of the centralized treatment scenarios are higher than the decentralized treatment scenarios. The aquatic eutrophication of the centralized treatment scenarios is lower than the decentralized treatment scenarios. This is attributable to lower total phosphorus in the effluent of the centralized treatment scenarios (0.73 g total P per m^3^ treated wastewater), compared to that of the decentralized treatment scenarios (1.52 g total P per m^3^ treated wastewater). In comparison with dewatering, sludge decomposition (i.e., for fertilizer) generates lower environmental impacts. According to Seleiman et al.^28^,Kominko et al.^41^, sludge is rich in nutrients that are beneficial for crop growth without contaminating groundwater and agriculture produce. However, in this current research, the heavy metals in sludge fertilizer, including copper, cadmium and mercury, exceed the regulatory limits on organic fertilizer standards^42^. To minimize food-related toxicity in human, the authorities thus stipulate that sludge fertilizers should be used in ornamental plants (Department of Drainage and Sewerage^24^.

**Table 2:** Environmental impacts and process contribution analysis of the centralized and decentralized sludge treatment scenarios.

Impact category	Unit	Centralized WWTPs			Decentralized WWTPs		
		C-dewatering	C-fertilizer	Process contributor	D-dewatering	D-fertilizer	Process contributor
Human toxicity, carcinogens	kg C_2_H_3_Cl-eq	0.0264	0.0260	Electricity use	0.0095	0.0091	Electricity use
Human toxicity, non- carcinogens	kg C_2_H_3_Cl-eq	0.0183	0.0053	Heavy metal in sludge	0.0063	0.0057	Heavy metal in sludge
Aquatic ecotoxicity	kg TEG-eq w	100.4296	90.8305	Heavy metal in sludge	87.1171	83.6021	Heavy metal in sludge
Terrestrial ecotoxicity	kg TEG-eq s	0.2130	0.1517	Electricity use	0.0791	0.0251	Electricity use
Global warming, fossil	kg CO_2_-eq	0.5340	0.4912	Electricity use	0.2496	0.2118	Electricity use
Respiratory organics	pers*ppm*h	1.40 × 10^–4^	1.27 × 10^–4^	Electricity use	7.60 × 10^–5^	6.48 × 10^–5^	Electricity use
Respiratory inorganics	kg PM_2.5_-eq	2.97 × 10^–4^	2.51 × 10^–4^	Electricity use	1.09 × 10^–4^	6.84 × 10^–5^	Electricity use
Photochemical ozone, vegetat	m2-years agr	1.6697	1.5072	Electricity use	1.5892	0.8152	Electricity use
Acidification	m^2^ UES	0.0230	0.0193	Electricity use	0.0211	0.0083	Electricity use
Aquatic eutrophication	kg NO_3_-eq	0.0398	0.0395	Effluent quality	0.0397	0.0723	Effluent quality
Terrestrial eutrophication	m^2^ UES	0.0254	0.0194	Electricity use	0.0224	0.0092	Electricity use
Nature occupation	m^2^-years agr	0.0028	0.0013	Electricity use	0.0020	0.0010	Electricity use
Non-renewable energy	MJ extra	7.6694	7.3235	Electricity use	7.4928	2.7355	Electricity use
Mineral extraction	m^2^-years agr	3.92 × 10^–5^	3.10 × 10^–6^	Electricity use	2.07 × 10^–5^	− 1.12 × 10^–5^	Tap water

**Figure 3:**
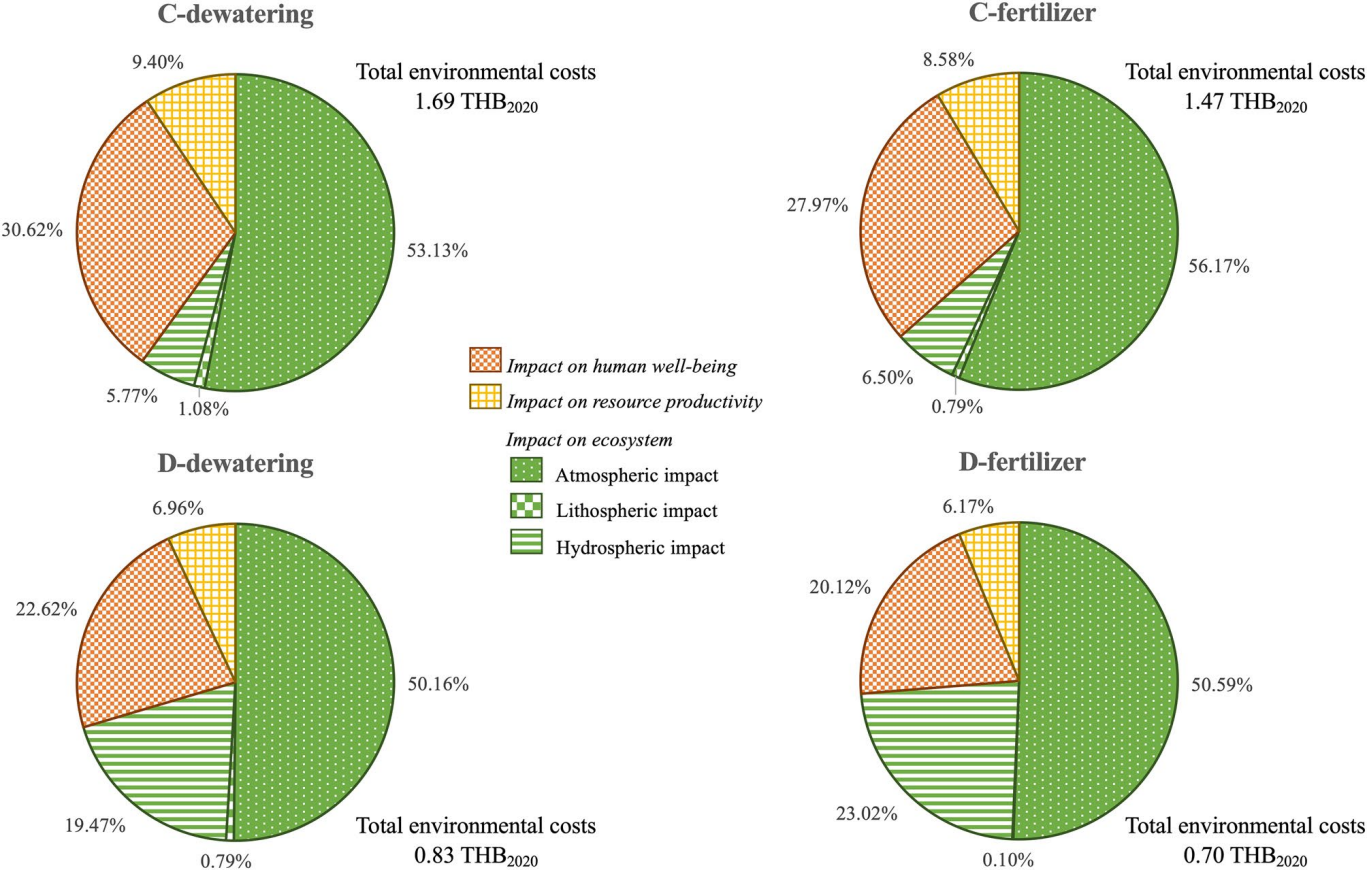
Total environmental costs and three damage categories of four sludge management scenarios.

Figure 3 compares the total environmental costs of four sludge management scenarios (C-dewatering, C-fertilizer, D-dewatering, D-fertilizer). The total environmental cost of the C-dewatering scenario is highest (1.69 THB_2020_ per m^3^ treated effluent), while that of the D-fertilizer scenario is lowest (0.70 THB_2020_ per m^3^ treated effluent). Of all the four scenarios, impact on ecosystem accounts for the largest proportion of the environmental costs (0.52–1.01 THB_2020_ per m^3^ treated effluent or 59.98–73.71% of total environmental costs) and the largest proportion of the impact on ecosystem is the atmospheric impact (0.35–0.90 THB_2020_ per m^3^ treated effluent or 50.16–56.17% of total environmental costs). The impacts on ecosystem and human well-being of all scenarios cover more than 90% of total environmental costs. The total environmental costs of the centralized sludge treatment scenarios (C-dewatering and C-fertilizer) are higher than the decentralized sludge treatment scenarios (D-dewatering and D-fertilizer). The total environmental costs of the dewatering scenarios (1.69 and 0.83 THB_2020_ per m^3^ treated effluent for C-dewatering and D-dewatering) are higher than those of C- and D-fertilizer scenarios (1.47 and 0.70 THB_2020_ per m^3^ treated effluent).

The sensitivity analysis showed negligible differences in the environmental impacts between the C- and D-dewatering scenarios, given the reduction in electricity consumption by 10%, 20%, and 30%. For the C- and D-fertilizer scenarios, heavy metals, nitrogen and phosphorus in sludge affect human toxicity, ecotoxicity, eutrophication and resources categories under Stepwise2006, CML-IA baseline, and ReCiPe methods. The findings are consistent with Heimersson et al^43^,Niero et al^44^,Renou et al.^45^.

### LCCA outcomes and the most cost-effective scenarios

**Figure 4:**
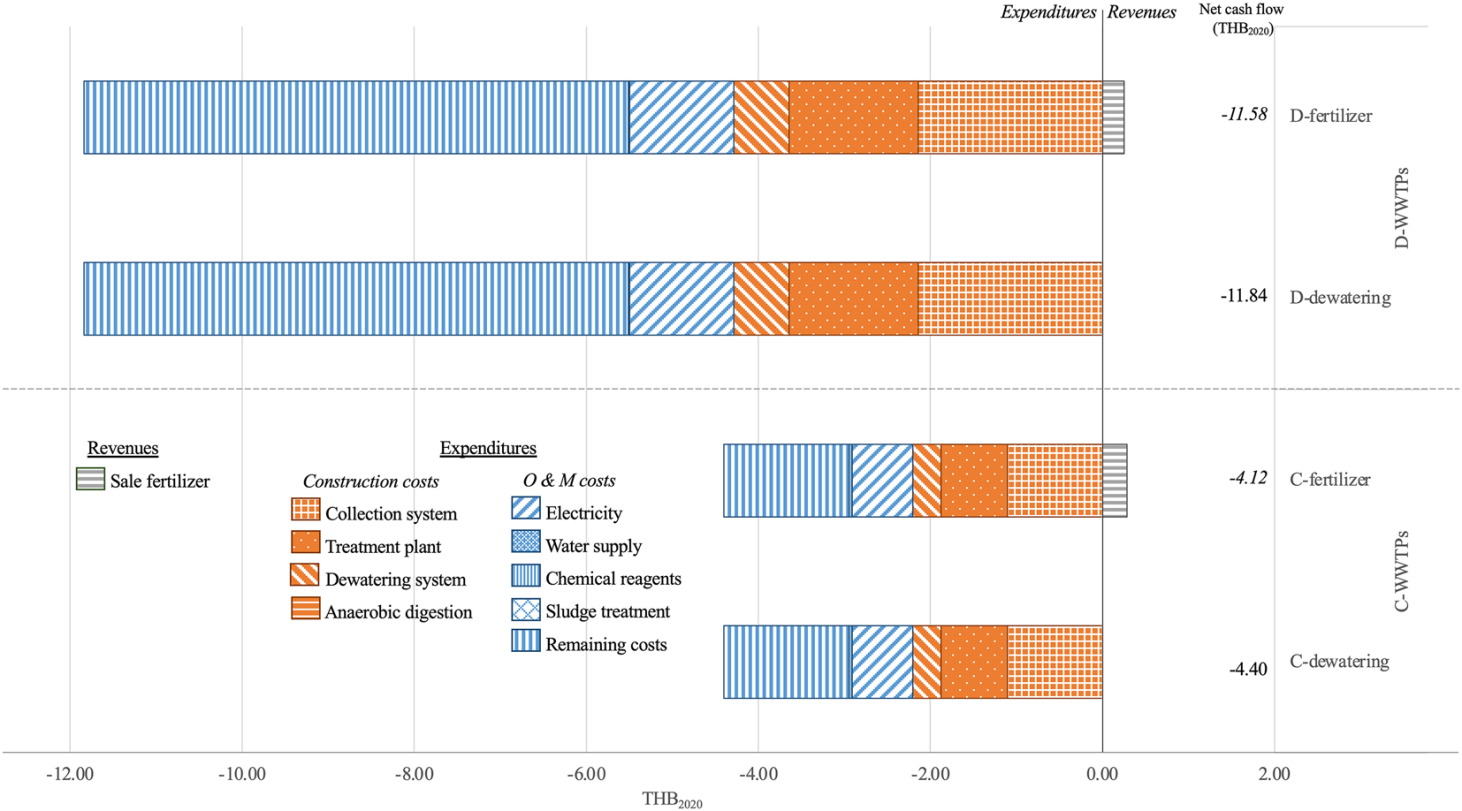
Cash inflow and cash outflow of four sludge treatment scenarios in Thai baht_2020_.

Figure 4 presents the revenue and expenditures of the four sludge management scenarios (C-dewatering, C-fertilizer, D-dewatering, D-fertilizer). The revenue from sale of sludge fertilizer under the C- and D-fertilizer scenarios are 0.29 and 0.25 THB_2020_ per m^3^ treated effluent. For the expenditures, the construction and O&M costs of the centralized treatment scenarios (2.21 and 2.20 THB_2020_ per m^3^ treated effluent) are lower than those of the decentralized treatment scenarios (4.28 and 7.55 THB_2020_ per m^3^ treated effluent). In this study, the total financial costs of the centralized sludge treatment scenarios (C-dewatering and C-fertilizer) are higher than the decentralized sludge treatment scenarios (D-dewatering and D-fertilizer). The finding however contradicts Jung et al.^3^.

The construction costs of the existing decentralized treatment scenarios are higher than the centralized treatment scenarios since most of the existing decentralized WWTPs in Thailand were constructed more than three decades and have treated wastewater using energy-inefficient technology, e.g., mechanical aerations^46^. The decentralized treatment scenarios are classified by the demand for electricity as the small general service and the centralized treatment scenarios as the large general service^24^. The electricity cost (THB per kWh) of the small general service (or the decentralized treatment scenarios) of 1.21 THB_2020_ per m^3^ treated effluent was higher than that of the large general service (or the centralized treatment scenarios) of 0.70 THB_2020_ per m^3^ treated effluent^47^. The administrative overheads, e.g., wage, management fee, of the decentralized treatment scenarios (6.33 THB_2020_ per m^3^ treated effluent) are higher than the centralized treatment scenarios (1.46 THB_2020_ per m^3^ treated effluent).

**Figure 5:**
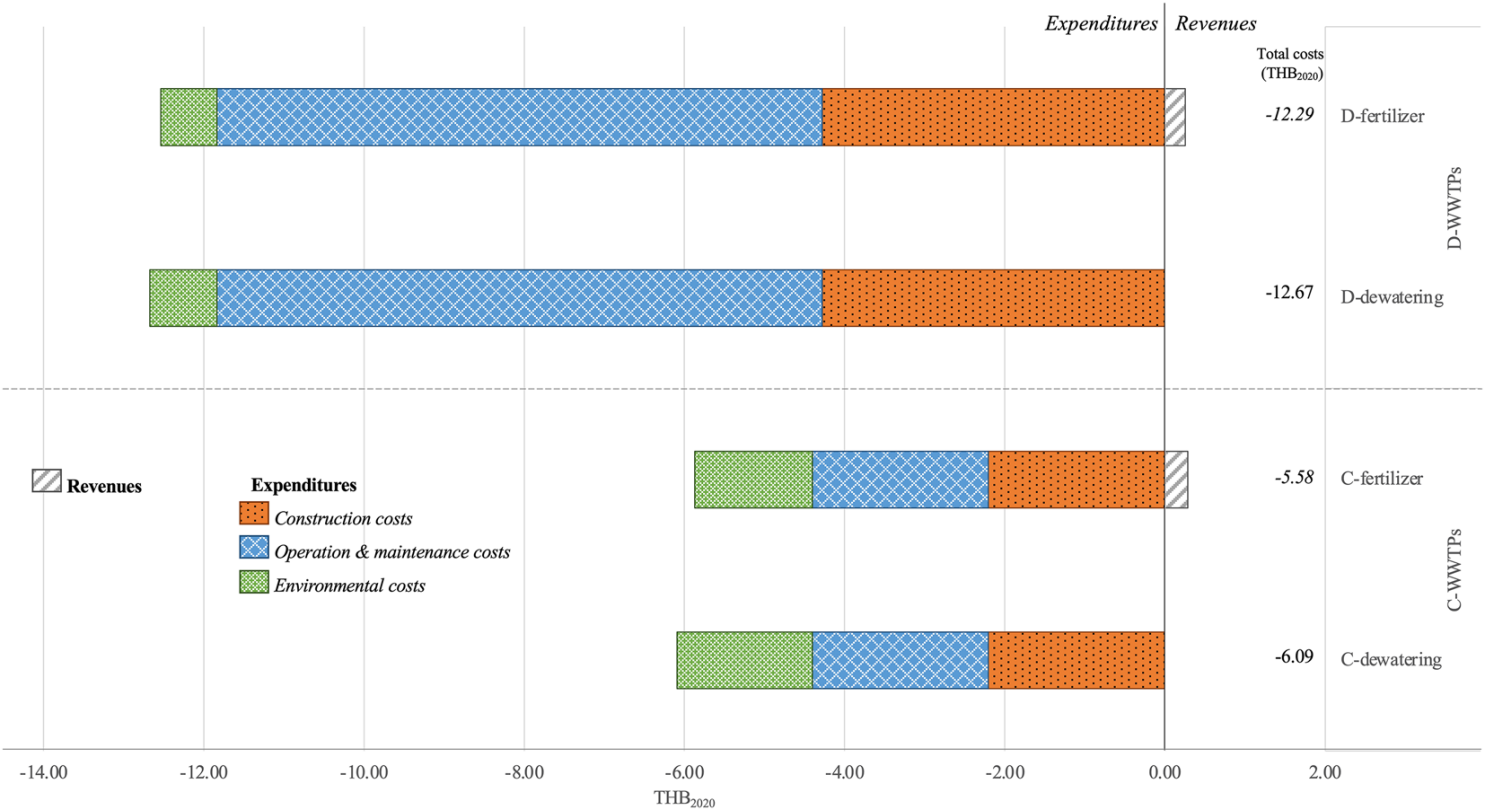
LCCA of four sludge treatment scenarios in Thai baht_2020_.

Figure 5 shows the LCCA results of the four sludge management scenarios (C-dewatering, C-fertilizer, D-dewatering, D-fertilizer). The LCCA of the centralized treatment scenarios (C-dewatering and C-fertilizer) (−6.09 and −5.58 THB_2020_ per m^3^ treated effluent, respectively) are higher than that of the decentralized scenarios (D-dewatering and D-fertilizer) (−12.67 and −12.29 THB_2020_ per m^3^ treated effluent, respectively). The LCCA of C- and D-fertilizer scenarios (−5.58 and −12.29 THB_2020_ per m^3^ treated effluent, respectively) are higher than those of the C- and D-dewatering scenarios (−6.09 and −12.67 THB_2020_ per m^3^ treated effluent, respectively). The O&M costs of the decentralized treatment scenarios (D-dewatering and D-fertilizer) account for 59.61–60.24% of total cash outflow (i.e., the construction (33.80–34.16%), environmental (5.60–6.59%) and O&M costs), unlike the centralized treatment scenarios (C-dewatering and C-fertilizer) in which the construction, O&M and environment costs account for 36.23–37.59%, 36.06–37.41%, and 25–27.71% of the total cash outflow, respectively.

For the most cost-effective sludge treatment scenarios, the C-fertilizer scenario is the most environmentally and economically viable scenario given the highest revenue and lowest total cash outflow. By comparison, the environmental costs of sludge decomposition (C- and D-fertilizer scenarios) are lower than those of dewatering (C- and D-dewatering scenarios). Meanwhile, the net cash flows of sludge decomposition are greater than those of dewatering.

### The outcomes of planned WWTPs

**Figure 6:**
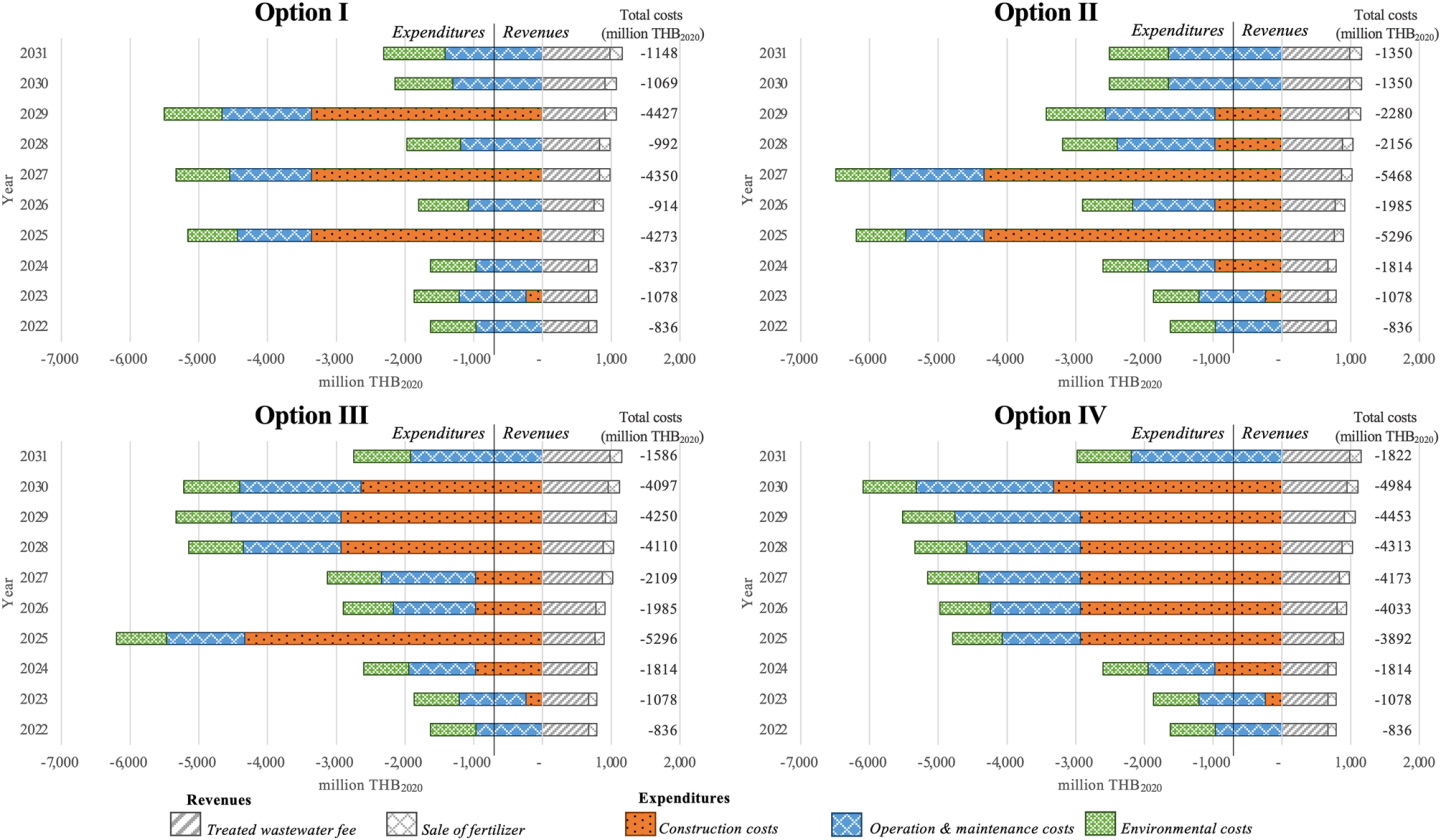
LCCA of the four WWTP construction options in million Thai baht_2020_.

Figure 6 shows the LCCA results of the four WWTP construction options (i.e., options I, II, III, and IV) for the period of 2022–2031. The annual total costs of the four options are negative because of higher cash outflow and environmental costs, in comparison with the cash inflow. The LCCA deficit of option I is lowest (-19,925 million THB_2020_), followed by option II (-23,613 million THB_2020_).

The NPVs of the four WWTP construction options are provided in Table SI-5 to SI-8 of SI. The negative NPVs are attributable to lower cash inflow (revenues), vis-à-vis the cash outflow (expenditures). The financial loss, as measured by the NPV, of option I is smallest (-6309.96 million THB_2020_), followed by option II (-6938.15 million THB_2020_). Meanwhile, the current wastewater treatment fee for Bangkok residents of 2 THB_2020_ per m^3^ wastewater is 3.5 times below the required minimum fee.

The WWTP construction option I, which entails building four centralized WWTPs within 10 years between 2022–2031, is the most environmentally and economically optimal WWTP construction option for the capital Bangkok, given its lowest LCCA deficit (−19925 million THB_2020_) and smallest financial loss (NPV = −6309.96 million THB_2020_).

## Conclusions

The research findings reveal that electricity consumption is the main contributor of almost environmental impacts in all four sludge treatment scenarios. All environmental impacts, excluding aquatic eutrophication, of the decentralized treatment scenarios are lower than those of the centralized treatment scenarios. The total environmental cost of the D-fertilizer scenario is lowest (0.70 THB_2020_ per m^3^ treated effluent), while that of the C-dewatering scenario is highest (1.69 THB_2020_ per m^3^ treated effluent). The largest proportion of the environmental costs is the impact on ecosystem of all the four scenarios (59.98–73.71% of total environmental costs). The environmental costs of the decentralized treatment scenarios are lower than those of the centralized treatment scenarios. The total environmental costs of C- and D-fertilizer scenarios are lower than those of C- and D-dewatering scenarios.

The total financial costs of the centralized treatment scenarios (C-dewatering and C-fertilizer) are greater than those of the decentralized treatment scenarios (D-dewatering and D-fertilizer). The construction and O&M costs of the decentralized treatment scenarios are higher than the centralized treatment scenarios. The LCCA deficit (including revenue, expenditures and environmental costs) of the C-fertilizer scenario is the smallest. Besides, the C-fertilizer scenario is the most environmentally and economically viable treatment scenario given its highest revenue and lowest expenditures. Specifically, composting should be adopted for sludge treatment.

The environmentally and financially optimal WWTP construction option is to construct four centralized WWTPs between 2022–2031 (option I), given the lowest LCCA deficit (−19925 million THB_2020_) and smallest financial loss (NPV = −6309.96 million THB_2020_). Essentially, the Bangkok Metropolitan Administration, which is the local government of the capital Bangkok, should adopt option I as a guideline in formulating the wastewater treatment management policy of 2022–2031.

## Supplementary Information

Supplementary Information.

## Data Availability

The authors declare that the inventory data and the findings of this study are available within the article and in the supplementary information.
