# Supplementary material for: Life cycle impact assessment and life cycle cost assessment for centralized and decentralized wastewater treatment plants in Thailand
Source: Sci Rep. 2022 Aug 25;12:14540. doi: 10.1038/s41598-022-18852-y (PMC9411131; doi:10.1038/s41598-022-18852-y)
Supplement: Supplementary file 1 — Supplementary Information. [file 41598_2022_18852_MOESM1_ESM.pdf]

Supplementary Information

Life cycle impact assessment and life cycle cost assessment for centralized and decentralized wastewater treatment plants in Thailand

Rutjaya Prateep Na Talang

Address: Department of Environmental Engineering, Faculty of Engineering, Kasetsart University, Bangkok 10900, Thailand

Phone: +662 7970999

Fax: +662 5790730

Email: rutjaya@gmail.com

Sanya Sirivithayapakorn

Address: Department of Environmental Engineering, Faculty of Engineering, Kasetsart University, Bangkok 10900, Thailand

Phone: +662 7970999

Fax: +662 5790730

Email: fengsys@ku.ac.th

Sucheela Polruang*

Address: Department of Environmental Engineering, Faculty of Engineering, Kasetsart University, Bangkok 10900, Thailand

Phone: +662 7970999

Fax: +662 5790730

Email: fengslpo@ku.ac.th

*corresponding author

Table SI-1 Life cycle inventory data of centralized and decentralized WWTPs

Data	Unit	Centralized WWTP		Decentralized WWTP	
		Average	Standard deviation	Average	Standard deviation
**Input**					
Electricity	kWh	0.873	0.321	0.363	0.161
Transport polymer	tkm	1.85×10^{-6} *		1.73×10^{-6}	8.57×10^{-9}
Polymer	kg	9.24×10^{-5}	8.96×10^{-5}	0.00009*	
Water supply	m^3	0.00625	0.00630	0.00544	0.00002
**Output**					
Wastewater	m^3	1.000		1.000	
Sludge	kg	0.0927	0.1018	0.0881	0.0008
Emission to air					
Carbon dioxide	kg	0.01896	0.00487	0.03662	0.01404
Methane	kg	0.00041	0.00052	0.00114	0.00134
Dinitrogen oxide	kg	0.00008	0.00005	0.00007*	
Emission to water					
BOD5	kg	0.00484	0.00157	0.00816	0.00248
COD	kg	0.02352	0.00730		
Suspended solids	kg	0.00695	0.00272	0.01136	0.00428
Phosphorus, total	kg	0.00073	0.00035	0.00152*	
Nitrogen, total	kg	0.00589	0.00207		
Nitrogen, TKN	kg			0.00586	0.00001

Note: * calculated amount from secondary data.

Table S-2 The midpoint impact categories of the Stepwise2006

Midpoint impact category	Unit	Category indicator	Characterization model source
Human toxicity (carcinogens),	kg C_2H_3Cl-eq	kg-equivalents of chloroethylene emitted into air	IMPACT2002+
Human toxicity (non- carcinogens)	kg C_2H_3Cl-eq	kg-equivalents of chloroethylene emitted into air	Added factors for 'aluminium, ion' emissions
Aquatic ecotoxicity,	kg-eq. TEG water	kg-equivalents triethylene glycol into water	IMPACT2002+
Terrestrial ecotoxicity	kg-eq. TEG soil	kg-equivalents triethylene glycol into soil	IMPACT2002+, To avoid double-counting with the nature occupation, the more localized impacts of emissions to soil are excluded in the Stepwise method.
Global warming	kg CO_2-eq	100 years time horizon	IPPC 2001 (also used in EDIP2003)
Respiratory inorganics	kg $PM_{2.5}$-eq	kg-equivalents of PM2.5 into air	IMPACT2002+
Respiratory organics	pers*ppm*h	person*ppm*hours (i.e., the product of the number of people exposed above the 60 ppb threshold, the annual duration of the exposure above the threshold, and the accumulated hourly mean ozone concentration over the threshold)	EDIP2003
Photochemical ozone	m^2*ppm*hours	m^2*ppm*hours (i.e., the product of the area of vegetation exposed to the 40 ppb threshold of chronic effects, the annual duration of exposure over the threshold, and the accumulated hourly mean ozone concentration over the threshold during daylight hours in the vegetation period)	EDIP2003
Acidification	m^2 UES	m^2 unprotected ecosystem	EDIP2003
Aquatic eutrophication	kg NO_3-eq	kg nitrate equivalents	New; developed for this method
Terrestrial eutrophication	m^2 UES	m^2 unprotected ecosystem (i.e. the ecosystem area that is brought to exceed the critical load for terrestrial eutrophication)	EDIP2003
Nature occupation	m^2-years agr	m^2-equivalents arable land (representing the impact on biodiversity from the occupation of one m^2 of arable land during one year)	modified from IMPACT2002+
Non-renewable energy	MJ primary	MJ total primary non-renewable energy	IMPACT2002+
Mineral extraction	MJ extra	MJ additional energy (the difference between the current energy requirement for extraction and an estimated future energy requirement for extraction from lower grade ores)	IMPACT2002+

Source: Weidema B P, et al. [1]

Table S-3 The monetary weighting factors in the Stepwise2006 method

Impact category		Unit	Monetary weighting factors ($Euro_{2003}$/unit)
Endpoint	Human well-being	QALY	74,000
	Ecosystem	specie	30,800,000,000
	Ecosystem	BAHY	1,400
Midpoint	Human toxicity (carcinogens),	kg C_2H_3Cl-eq	0.27
	Human toxicity (non-carcinogens)	kg C_2H_3Cl-eq	0.27
	Aquatic ecotoxicity,	kg-eq. TEG water	0.0000071
	Terrestrial ecotoxicity	kg-eq. TEG soil	0.0011
	Global warming	kg CO_2-eq	0.083
	Respiratory inorganics	kg $PM_{2.5}$-eq	68
	Respiratory organics	pers*ppm*h	0.26
	Photochemical ozone	m^2*ppm*hours	0.00037
	Acidification	m^2 UES	0.0077
	Aquatic eutrophication	kg NO_3-eq	0.10
	Terrestrial eutrophication	m^2 UES	0.013
	Nature occupation	m^2-years agr	0.12
	Non-renewable energy	MJ primary	-
	Mineral extraction	MJ extra	0.004

Source: Weidema B P [2], Pizzol, et al. [3]

Note: Quality Adjusted Life Year (QALY); Biodiversity Adjusted Hectare Year (BAHY)

Currency conversation equations

In environmental cost analysis, the weighting results in the 2003 European currency (EUR_{2003}) were converted into the 2003 Thai currency (THB_{2003}) based on purchasing power parity (PPP) [4], as expressed in Equation (1) [5]. The conversion of year-value from 2003 to 2020 using gross domestic product (GDP) deflator index [4] was calculated by equation (2) [5]. The conversion factors are tabulated in Table SI-4.

$$THB_{2003} = \frac{EUR_{2003} \times PPP_{THB2003}}{PPP_{EUR2003}}, \quad (1)$$

where THB_{2003} and EUR_{2003} are the 2003 Thai baht (THB) and Euro (EUR) values, and $PPP_{THB2003}$ and $PPP_{EUR2003}$ are the exchange rate-adjusted purchasing power parity for Thai baht (THB) and Euro (EUR).

$$THB_{2020} = \frac{THB_{2003} \times D_{2020}}{D_{2003}} \quad (2)$$

where THB_{2003} and THB_{2020} are the 2003 and 2020 Thai baht (THB) values, and D_{2003} and D_{2020} are the 2003 and 2020 GDP deflator indexes.

Table SI-4 The conversion factors for LCCA

List	Conversion factor	Reference
$PPP_{Thai2003}$	11.04	World Bank [4]
$PPP_{EUR2003}$	0.84	OECD [6]
$GDP_{Thai2003}$	102.15	World Bank [4]
$GDP_{Thai2020}$	158.35	World Bank [4]

Table SI-5 Annual cash flow and the net present value (NPV) of option I where the 10-year period corresponds to the years 2022 – 2031 in million Thai baht$_{2020}$.

Year	Revenues		Expenditures		Period	Cash flow
	Treated WW fee	Sale of fertilizer	O&M costs	Environmental costs		
2022	669.85	119.08	-968.29	-656.91	0	-836.27
2023	669.85	119.08	-968.29	-657.23	1	-836.59
2024	669.85	119.08	-968.29	-657.47	2	-836.80
2025	751.02	133.55	-1079.70	-719.16	3	-914.29
2026	751.02	133.55	-1079.70	-719.28	4	-914.41
2027	832.20	148.02	-1191.12	-781.02	5	-991.92
2028	832.20	148.02	-1191.12	-781.00	6	-991.86
2029	913.38	162.48	-1302.54	-842.52	7	-1069.20
2030	913.38	162.48	-1302.54	-842.29	8	-1068.98
2031	985.88	176.95	-1413.96	-897.12	9	-1148.24
	Discount Rate		10%		NPV	-6309.96

Table SI-6 Annual cash flow and the net present value (NPV) of option II where the 10-year period corresponds to the years 2022 – 2031 in million Thai baht$_{2020}$.

Year	Revenues		Expenditures		Period	Cash flow
	Treated WW fee	Sale of fertilizer	O&M costs	Environmental costs		
2022	669.85	119.08	-968.29	-656.91	0	-836.27
2023	669.85	119.08	-968.29	-657.23	1	-836.59
2024	669.85	119.08	-968.29	-657.45	2	-836.80
2025	763.19	135.46	-1137.14	-722.59	3	-961.07
2026	775.36	137.38	-1194.57	-726.13	4	-1007.97
2027	868.70	153.76	-1363.42	-791.30	5	-1132.26
2028	880.87	155.68	-1420.86	-794.66	6	-1178.97
2029	974.21	172.06	-1589.71	-859.65	7	-1303.09
2030	986.38	173.98	-1647.14	-862.85	8	-1349.64
2031	985.88	173.98	-1647.14	-862.41	9	-1349.69
	Discount Rate		10%		NPV	-6938.15

Table SI-7 Annual cash flow and the net present value (NPV) of option III where the 10-year period corresponds to the years 2022 – 2031 in million Thai baht$_{2020}$.

Year	Revenues		Expenditures		Period	Cash flow
	Treated WW fee	Sale of fertilizer	O&M costs	Environmental costs		
2022	669.85	119.08	-968.29	-656.91	0	-836.27
2023	669.85	119.08	-968.29	-657.23	1	-836.59
2024	669.85	119.08	-968.29	-657.45	2	-836.80
2025	763.19	135.46	-1137.14	-722.59	3	-961.07
2026	775.36	137.38	-1194.57	-726.13	4	-1007.97
2027	868.70	153.76	-1363.42	-791.30	5	-1132.26
2028	880.87	155.68	-1420.86	-794.66	6	-1178.97
2029	917.37	161.42	-1593.16	-804.79	7	-1319.16
2030	953.87	167.17	-1765.46	-814.84	8	-1459.26
2031	985.88	172.34	-1920.53	-823.56	9	-1585.86
	Discount Rate		10%		NPV	-7097.69

Table SI-8 Annual cash flow and the net present value (NPV) of option IV where the 10-year period corresponds to the years 2022 – 2031 in million Thai baht$_{2020}$.

Year	Revenues		Expenditures		Period	Cash flow
	Treated WW fee	Sale of fertilizer	O&M costs	Environmental costs		
2022	669.85	119.08	-968.29	-656.91	0	-836.27
2023	669.85	119.08	-968.29	-657.23	1	-836.59
2024	669.85	119.08	-968.29	-657.45	2	-836.80
2025	763.19	135.46	-1137.14	-722.59	3	-961.07
2026	799.69	141.21	-1309.44	-732.98	4	-1101.52
2027	836.19	146.96	-1481.74	-743.29	5	-1241.88
2028	872.69	152.70	-1654.04	-753.50	6	-1382.15
2029	909.19	158.45	-1826.34	-763.63	7	-1522.34
2030	945.69	164.19	-1998.64	-773.68	8	-1662.44
2031	985.88	170.71	-2193.92	-784.70	9	-1822.03
	Discount Rate		10%		NPV	-7643.56

References

1 Weidema B P, Wesnæs M, Hermansen J, Kristensen T & Halberg N. in *Environmental improvement potentials of meat and dairy products* (eds Eder P & Delgado L) (Institute for Prospective Technological Studies, Joint Research Centre, Sevilla, Spain, 2008).

2 Weidema B P. in *Environmental improvement potentials of meat and dairy products* (eds Eder P & Delgado L) (Institute for Prospective Technological Studies, Sevilla, Spain, 2008).

3 Pizzol, M., Weidema, B., Brandão, M. & Osset, P. Monetary valuation in Life Cycle Assessment: a review. *J. Clean. Prod.* **86**, 170-179, doi:10.1016/j.jclepro.2014.08.007 (2015).

4 World Bank. *World Databank*, <data.worldbank.org> (2020).

5 Brander, L. (Ecosystem Services Economics Unit, UNEP-DEPI, Nairobi, Kenya, 2013).

6 OECD. *Purchasing power parities (PPP)*, <www.oecd.org> (2018).
